# Consequences and Utility of the Zinc-Dependent Metalloprotease Activity of Anthrax Lethal Toxin 

**DOI:** 10.3390/toxins2051038

**Published:** 2010-05-11

**Authors:** Jennifer Bromberg-White, Chih-Shia Lee, Nicholas Duesbery

**Affiliations:** 1 Laboratory of Cancer and Developmental Cell Biology, The Van Andel Research Institute, 333 Bostwick NE Grand Rapids, MI, 49503, USA; Email: jenn.white@vai.org (J.B.-W.); jenn.white@vai.org (C.-S.L.); 2 Department of Biochemistry and Molecular Biology, Michigan State University, East Lansing MI 48824, USA

**Keywords:** anthrax, lethal factor, mitogen-activated protein kinase kinase, pathogenesis, metalloprotease, tumorigenesis, retinal neovascularization

## Abstract

Anthrax is caused by the gram-positive bacterium *Bacillus anthracis*. The pathogenesis of this disease is dependent on the presence of two binary toxins, edema toxin (EdTx) and lethal toxin (LeTx). LeTx, the major virulence factor contributing to anthrax, contains the effector moiety lethal factor (LF), a zinc-dependent metalloprotease specific for targeting mitogen-activated protein kinase kinases. This review will focus on the protease-specific activity and function of LF, and will include a discussion on the implications and consequences of this activity, both in terms of anthrax disease, and how this activity can be exploited to gain insight into other pathologic conditions.

## 1. Introduction

Over the last decade there has been a renewed interest in understanding anthrax due to the recent use of anthrax as a biological weapon. While the mechanism by which anthrax kills its host is still unclear, the lethality of the disease was attributed to the production of anthrax toxin more than 50 years ago [1]. Following this seminal discovery by Smith and Keppie, it has been determined that anthrax toxin is composed of three proteins that when combined in pairwise fashion form two binary toxins: protective antigen (PA), edema factor (EF), and lethal factor (LF) [2]. PA alone is non-toxic, but serves to translocate EF or LF to the cytosol [3]. The combination of PA with EF, called edema toxin (EdTx), results in edema upon subcutaneous injection, but is nontoxic following intravenous administration to animals [4]. PA plus LF does not induce edema, however is toxic when injected intravenously into animals [4,5], and is therefore referred to as lethal toxin (LeTx) [5]. 

Prior to EF or LF translocation to the cytosol, PA must first bind to one of two identified cell surface anthrax toxin receptors, tumor endothelial marker-8 (TEM8) and capillary morphogenesis gene-2 (CMG2)[6,7]. Proteolytic cleavage of PA by cell surface-associated furin generates an active molecule, which forms a heptameric prepore complex capable of binding up to three EF and/or LF molecules (although evidence exists of cleaved PA in the circulation [8]). Upon endocytosis via the endosomal pathway, changes in pH alter prepore formation, allowing translocation of EF and LF to the cytosol where they perform their enzymatic functions [9]. 

EF is a calmodulin-dependent adenylate cyclase that upon activation increases the conversion of intracellular ATP to cyclic AMP (cAMP). This results in the disruption of water homeostasis followed by edema [10]. Although *B. anthracis* strains deficient in EF production were shown to still be lethal in mice [11], EF does in fact play a role in anthrax pathogenesis. EdTx can inhibit phagocytosis of *B. anthracis* by neutrophils [12], suggesting that EdTx can increase susceptibility to infection by suppressing neutrophil function. Furthermore, EdTx has been shown to be cytotoxic, causing tissue necrosis using a zebrafish model [13], as well as tissue damage and lethality in mice [14], implicating EdTx as having a toxic role in anthrax pathogenesis.

LeTx is considered the major virulence factor of anthrax and the mediator of host lethality. Consequently, research efforts have focused more heavily on the mechanism of action of LeTx compared to that of EdTx. Vascular pathologies such as hemorrhage and septic shock are common features of anthrax, as is modulation of the host immune system, and LeTx is thought to play a causative role in these pathologies [15,16,17]. Furthermore, LeTx can induce macrophage apoptosis [18], modulate cytokine secretion of dendritic cells [19], and directly inhibit T-cell activation [20]. LeTx has been shown to play a role in vascular integrity and endothelial cell function in clinical examples [21] and experimental models [22,23,24,25]. However, the mechanism of anthrax lethality is still not understood. In this review, we will describe the identification of LF as a zinc-dependent metalloprotease specific for mitogen activated protein kinase kinases (MAPKKs, MEKs or MKKs), and the potential consequences of this activity to the host following anthrax infection in terms of disease pathogenesis. Finally, we will explore the utility of exploiting this activity to further our understanding not only of the mechanism of LeTx action, but also as a tool to evaluate the role of MEK signaling pathways in neovascular disease.

## 2. Structure and Function of Lethal Factor

### 2.1. Identification of functional domains of LF

Lethal factor (LF) is a 90 kDa secreted protein encoded by the *lef* locus on the pXOI plasmid of *B. anthracis* [26]. The crystal structure of LF has been solved and is reviewed elsewhere [27]. LF is encoded by a 2,427 bp open reading frame that can be divided into three regions: (1) a PA-binding region contained within the first 254 amino acids at the NH_2_ terminus, which has a high degree of similarity to the amino terminus of EF, (2) a central region containing a series of five imperfect 19 amino acid repeats, and (3) the remaining C-terminal portion of the protein that exhibits no sequence homology to known proteins (reviewed in [28]). Mutagenesis mapping of LF demonstrated that the C-terminal region is responsible for the enzymatic activity of the protein. Insertions into this region eliminated toxicity without alteration in PA binding [29]. Further analysis of this domain identified a portion (amino acids 686-692; protein sequence HEFGHAV) containing a motif characteristic of metalloproteases (HEXXH, where X is any amino acid) [30].

### 2.2. LF is a zinc-dependent metalloprotease

The presence of a metalloprotease-like motif contained within the catalytic portion of LF suggested LF was a protease. In support of this, protease inhibitors such as bestatin and captopril blocked LF-mediated toxicity of macrophages [30]. Moreover, the substitution of alanine for two residues implicated in zinc binding (H686A and H690A) resulted in LF inactivation as well as reduced zinc binding, and substitution of cysteine for glutamic acid at amino acid 687 (E687C), a residue known to be essential for metalloprotease activity, led to the inactivation of LF [30]. Finally, LF has been shown to bind at least one ^65^Zn atom [30,31], and zinc binding is reduced in inactive LF mutants [30]. 

## 3. LF Zinc-Metalloprotease Activity Is Specific for the MAPK Pathway

While evidence strongly indicated that LF was a zinc-dependent metalloprotease, it was several years before the enzymatic substrate of LF was identified. In 1998, two groups independently identified mitogen-activated protein kinase kinase 1 and 2 (MAPPK1 and 2, or MEK1 and 2) as proteolytic substrates for LF [32,33]. 

**Figure 1:**
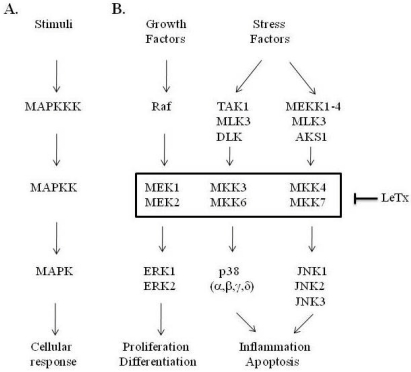
Schematic of the kinase cascade and resulting cellular responses of the MAPK signaling pathways. (A) Generic scheme of the MAPK signaling cascade, whereby an extracellular stimulus activates MAPKKK, which phosphorylates and activates MAPKK, which phosphorylates and activates MAPK, leading to an intracellular biological response. (B) Schematic of the specific MAPK factors within each of the three major MAPK pathways. LeTx targets the MAPKK tier in the cascade, cleaving and inactivating all the MAPKK (MEK1-2, MKK3-7) with the exception of MKK5, the pathway for which is not depicted.

The MAPK pathway is a key regulatory signal transduction pathway, which sends signals from the cell surface to intracellular effectors via a cascade of phosphorylation events (Figure 1). In mammalian cells, there are three extensively studied MAPK pathways: the extracellular signal-regulated kinase pathway (ERK), the p38 MAPK pathway (p38), and the c-Jun N-terminal kinase pathway (JNK). The ERK pathway is preferentially activated by growth factors, while the p38 and JNK pathways respond to cellular stresses such as osmotic shock and inflammatory cytokines (reviewed in [34]). ERK1 and ERK2 are activated by MEK1 and MEK2, p38 by MKK3 and MKK6, while JNK is activated by MKK4 and MKK7. These MAPKKs are in turn activated by different MAPKK kinases (MAPKKKs), which are differentially activated by extracellular stimuli, including growth factors, inflammatory signals, and environmental stresses (reviewed in [35]). These pathways regulate a variety of cellular responses including cell cycle progression, cell division, differentiation, motility, apoptosis, and survival [35]. 

**Figure 2:**
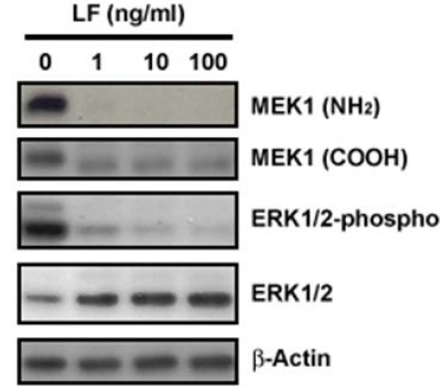
Inhibitory effect of LeTx on MEK signaling pathway in cells. Human melanoma SK-MEL-28 cells were treated with LeTx in a LF concentration-dependent manner (1 μg/mL PA plus 0, 1, 10, or 100 ng/mL LF) for 24 h. Whole cell extracts were then harvested and subjected to immunoblotting with antibodies against MEK1 N-terminus (top panel), MEK1 C-terminus (second panel), phospho-ERK1/2 (third panel), total ERK1/2 (fourth panel) and β actin (bottom panel).

That the MAPKKs were substrates for LF cleavage was first suggested by the observation of a subtle shift in electrophoretic mobility of MEK1 following LeTx treatment, which implicated a proteolytic modification of MAPKK by LF; this modification was subsequently shown to be located at the N-terminus of MAPKK [32,33]. It was quickly determined thereafter that LF cleaves all the MAPKK with the exception of MKK5 [36,37,38] (Figure 2). Interestingly, MKK4 and MKK7 appear to contain two cleavage sites within close proximity to each other in the N-terminal region, while the other MAPKKs contain single cleavage sites (Table 1). Analysis of the cleavage sites indicated a preferential cleavage just before an aliphatic residue, which is located two-to-three residues from a stretch of basic amino acids (Table 1). Generic MAPK binding sites (referred to as the MAPK docking domain) display a similar pattern of amino acids, consisting of a basic residue flanked by hydrophobic residues on one or both sides [39,40]. This provided a hint to a mechanism by which LF cleavage inactivates MAPKKs. In fact, LF was shown to reduce the affinity of MEK1 for MAPK [41,42], and decrease the intrinsic kinase activity of MEK [41]. Other regions of MEK besides the N-terminus appear to be required for LF-substrate recognition [33,41], which may explain the specificity of LF for MAPKKs and not other proteins containing a MAPK-interacting D domain.

**Table 1:** Alignment of MEK/MKK amino acid sequences flanking the LF cleavage sites. MEK/MKK amino acid sequences (single-letter codes) are aligned to the position where LF cleaves (slash). Aliphatic residues (Al) at the protease cleavage position 1’ (bold) are fully conserved in all the LF cleavage sites. The basic (B) or proline (P) residues prior to the cleavage sites are underlined. (X) variable residues.

**LF substrates**	**Amino acid sequences**	
MEK1	M PKKK P T P^(8)^	/ **I** Q L N P A P D
MEK2	A RRKP V L P^(11)^	/ **A** L T I N P T I
MKK3	S KRKK D L R^(26)^	/ **I** S C M S K P P
MKK4(K^45^-L^46^)	Q G KRK A L K^(45)^	/ **L** N F A N P P F
MKK4(R^58^-F^59^)	P P F K S T A R^(58)^	/ **F** T L N P N P T
MKK6	K KR N P G L K^(14)^	/ **I** P K E A F E Q
MKK7(Q^44^-L^45^)	Q RPRP T L Q^(44)^	/ **L** P L A N D G G
MKK7(Q^76^-L^77^)	A RPRH M L G^(76)^	/ **L** P S T L F T P
Consensus sequence	(B/P)_3-4_ X X X	/ **Al**

## 4. Consequences of the Zinc-Dependent Metalloprotease Activity of LF

### 4.1. Immune modulation

To establish a successful infection, *B. anthracis* must have mechanisms in place to suppress the immune system. LeTx affects various aspects of the immune system including cytokine, dendritic cell and T-cell responses [15,17]. LeTx appears to exert its immunosuppressive effects by blocking the function of phagocytes, resulting in a delay in wound healing and favoring bacterial growth, and by inhibiting cell-mediated immunity, to prevent death of infected macrophages. A well studied major cellular target of LeTx is macrophages, as LeTx was first demonstrated to exhibit cytotoxicity *in vitro* to murine macrophages [43]. It is now clear that macrophages are not the only target cell of LeTx, particularly in the immune system [44]. Monocytes, dendritic cells, and T cells, among others, all appear to be disregulated, in terms of cytokine secretion, activation, and proliferation, following LeTx treatment. In particular, LeTx is a potent T cell suppressor, both in terms of T cell activation and proliferation, but also in the ability of T cells to migrate and chemotax [45]. The inhibition of T cell chemotaxis likely impairs numerous pathways that contribute to bacterial clearance and wound healing.

The MAPK pathways are central to the activation of both the innate and adaptive immune responses [46]. MAPK activation has been suggested to play a role in macrophage phagocytosis [47,48]. Additionally, MAPK signaling pathways play key roles in activated T cell gene expression, and T cells disrupted in MAPK pathway signaling via LeTx-induced cleavage of MAPKKs have dramatic alterations in the activation of key transcription factors [20,49]. While, activation of the ERK pathway has been shown to be important in T cell maturation, activation, and differentiation [50,51], it is the p38 and JNK pathways that exert the greatest influences on immune responses. Activation of these pathways is important for a variety of immune responses, including initiation, activation, and progression of both the innate and adaptive immunities [52,53]. These pathways can regulate the expression of pro-inflammatory cytokines in macrophages [53], contribute to the development, activation, and proliferation of T cells [54,55], and play roles in dendritic cell migration and activation [56,57].

### 4.2. Vascular damage

Vascular damage and dysfunction are hallmarks of anthrax infection; vascular leakage, tissue hemorrhage, and terminal hypotensive shock are commonly associated with anthrax pathology. These effects are likely caused by increased vascular permeability. This has led to studies aimed at identifying potential direct effects of LeTx action on endothelial cells, and suggests that LeTx-mediated MAPKK inhibition may alter endothelial cell function. In support of this, *in vitro* studies have shown that LeTx can reduce endothelial cell viability and induces apoptosis of endothelial cells [22], as well as induce endothelial barrier dysfunction [23,58]. 

That alterations in vascular permeability and endothelial cell function result from MAPK pathway interference is not surprising. The MAPK pathway has been shown to play a pivotal role in vascularization in early embryonic development [59]. Deficiencies in various components of the MAPK cascade, as studied by way of knockout mice, result in defects in embryonic vascularization. For example, MEK1 and ERK2 knockout mice have defective placental vascularization [60,61], defects in MEK5 result in cardiovascular defects [62], and B-Raf knockout mice display extensive vascular defects [63]. 

## 5. The Utility of the MEK-Dependent Metalloprotease Activity of LF in Other Pathological Conditions

### 5.1. Tumor growth and angiogenesis

It is of interest to note that the initial discovery suggesting MAPKK as the substrate of LF came from a sensitivity screen of the National Cancer Institute’s anti-neoplastic drug screen database, which identified LF as having a similar sensitivity profile against 60 human cancer cell lines as the known MEK inhibitor, PD98059 [32,64]. Not only did this suggest MAPKKs as substrates for LF, but implicated LeTx as a potential novel therapeutic for tumorigenesis.

**Figure 3:**
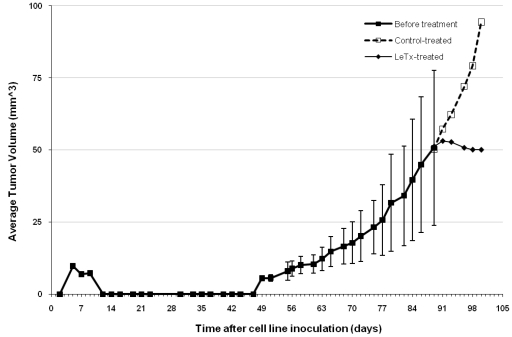
Inhibitory effect of LeTx on human melanoma SK-MEL-28 xenograft tumor growth. Human melanoma SK-MEL-28 cells were subcutaneously injected (10^7^ cells in 100 μl HBSS) into the right side of the dorsalateral area of athymic nude mice (10 mice per group). After tumors were established to a volume of 50 mm^3^, mice were intravenously injected with either LeTx (PA plus LF) or control (PA plus LF_E687C) at one standard dose (SD, 1 SD equals 10 μg PA plus 2 μg of LF or LF_E687C) every other day for a total of six injections. Tumor growth is presented as average tumor volume (mm^3^) against days following inoculation of tumor cells. (■) average volume of the total 10 tumors before treatment. (□) average volume of the tumors in control-treated mice. (♦) average volume of the tumors in LeTx-treated mice.

The MAPK signaling pathways have been intensely studied in terms of their roles in tumor growth and progression due to their implicated actions as regulators of cell proliferation, migration, and apoptosis, all of which are critical steps for tumor growth, survival, and metastasis [65]. In fact, MAPK pathways have been suggested to play critical roles in tumorigenesis based on the fact that they regulate proteolytic enzymes that can promote invasion and progression, migration and motility, which may enhance metastatic potential, and regulation of apoptosis to promote survival, particularly of metastatic cells to distant locations. Not surprisingly, therefore, LeTx was initially shown to inhibit the growth and tumorigenicity of V-12 H-*ras* transformed NIH 3T3 fibroblasts *in vitro* [38]. It has since been demonstrated that *invivo* administration of LeTx by intratumoral administration of LeTx [38,64], as well as by systemic treatment [66,67,68], inhibits tumor growth (Figure 3).

While systemic administration of LeTx reduced tumor growth in xenograft models, it also substantially reduced tumor vascular content, indicating that MAPKK signaling is important for vascularization of tumors *invivo* [38,67,68]. Importantly, as tumor vascularization is driven by the release of angio-proliferative growth factors from tumor cells that induce angiogenesis, analysis of tumor xenografts revealed that LeTx treatment decreased the release of a number of angio-proliferative factors, including basic fibroblast growth factor (bFGF), interleukin-8 (IL-8), and vascular endothelial growth factor (VEGF) [68], the same cytokines noted as strong correlates of disease-free and overall survival in other tumors such as melanoma [69]. 

Recently, PA alone has been reported to inhibit tumor angiogenesis [70]. However, these effects are only evident at relatively elevated concentrations, and by use of a mutant form of PA that is resistant to furin activation. At concentrations associated with LF-impaired angiogenesis (*i.e.*, 10-100 fold lower), wild-type PA alone or in combination with catalytically inactive LF has no measurable effect on angiogenesis [64,68]. Interestingly, the extracellular domain of anthrax toxin receptors (ANTXR) contains a von Willebrand factor type A (vWFA) domain, a conserved ligand binding that mediates extracellular matrix associations [71]. These domains within ANTXR have since been shown to associate with specific extracellular matrix proteins such as gelatin and type 1 collagen for TEM8 [72], and collagen type IV and laminin for CMG2 [73]. As PA-binding to these receptors could interfere with extracellular matrix interactions by ANTXR, elevated levels of extracellular PA may disrupt endothelial cell adhesion, leading to impaired angiogenesis.

MAPK pathway signaling has previously been shown to play essential roles in vascularization during tumorigenesis, specifically by modulating the release of and response to VEGF, which is recognized as a critical growth factor in angiogenesis [74]. MEK activity regulates VEGF expression at the transcriptional and post-transcriptional levels [75]. The ERK pathway has been demonstrated to be critical in the control of VEGF expression [76] and to mediate VEGF-induced proliferation via the endothelial-specific receptor, VEGFR2 [77], while JNK and p38 have been shown to regulate VEGF expression [75]. MEK signaling pathways are also activated in response to VEGF, whereby treatment of endothelial cells with VEGF results in activation of ERK1/2 [78], as well as p38 [79]. Insight into the *in vivo* functions of MAPKKs has been implicated by the effect of MAPKK inhibitors on tumor vascularization. For example, expression of an inactive Raf-1 mutant in endothelial cells blocks growth and vascularization of melanomas in mice [80], while BAY 43-9006 (Sorafenib), a compound that inhibits B-Raf and c-Raf, (MAPKKK isoforms that activate MEK1 and MEK2), also reduces tumor vascularization *in vivo* [81]. More recently, expression of dominant negative MKK1 in tumor endothelium has been shown to disrupt growth and vascularization of colorectal adenocarcinoma xenografts [82].

Whereas *in vitro* studies of LeTx pointed to a role for MAPK signaling in regulating VEGF expression in tumor cells, a different picture emerged from *in vivo* studies in which it was noted that tumors deficient in anthrax toxin receptor expression were still sensitive to LeTx treatment, resulting in decreased tumor growth *in vivo* [83]. Furthermore, vital imaging, performed on fibrosarcoma xenografts using high-resolution ultrasound, demonstrated that MEK inhibition through LeTx treatment led to a striking and rapid reduction in tumor perfusion within 24 h of LeTx treatment [68]. While its ability to reduce tumor vascularization may be linked to decreased tumor-cytokine production, the ability of LeTx to block growth of receptor deficient tumors, as well as the rapid reduction in perfusion following LeTx administration, strongly argues that MEK inhibition by LeTx inhibits tumor vascularization not by direct action on tumor cells, but through a stromal component of the tumor, perhaps endothelial cells.

### 5.2. Retinal Neovascularization

To further investigate the effects of LeTx on neovascularization, a mouse model of retinal vascular growth and neovascularization has been adapted [84]. Retinal vasculature forms in a well-characterized and highly reproducible manner, providing a convenient system to evaluate contributions to vascular formation and development. Due to vascular growth in two dimensions, the retina provides a convenient model to directly observe developmental angiogenesis, as well as a useful model to monitor and quantify changes in vascular growth. Retinal vascular development is intensely studied, especially in the context of retinopathies, whereby abnormal vascular growth (termed neovascularization) in the retina can lead to blindness. Common retinopathies of this nature include retinopathy of prematurity (ROP) and diabetic retinopathy (DR), which are characterized by retinal neovascularization [85], and age-related macular degeneration (AMD), which is characterized by pathological outgrowth of new vessels from the choroid into the subretinal space [86]. VEGF has been shown to play a central role as a stimulator of both retinal and choroidal neovascularization, whereby inhibition of VEGF can block both types of neovascularization in the eye [86,87,88]. In fact, intravitreal injections of agents that block VEGF function have been shown to stabilize and even potentially improve vision in patients with AMD [89]. 

Previously published reports suggest a role for the signaling of the MAPK pathways during both vascular development and disease progression in the retina. It has been demonstrated *in vitro* that while the Ras/Raf/MEK/ERK pathway activated proliferation of retinal pigmented epithelial (RPE) cells [90], the JNK and p38 MAPK pathways have been characterized for their role in RPE cell death [91]. Furthermore, an increase of ERK activation was detected in a rat model of ROP, and intravitreous injection of ERK inhibitors reduced retinal neovascularization in this *invivo* model system [92]. Increased MAPK activation has been reported in retinal ischemia-reperfusion models [93,94], and recently, the JNK pathway has been shown to play a key role in retinal neovascularization in a mouse model of ROP [95]. It has since been demonstrated that LeTx delays the sprouting angiogenesis and branching morphogenesis during developmental vascularization in the murine retina [84], and appears to inhibit both neovascularization and revascularization following oxygen-induced retinopathy [96]. These data suggest that MAPK signaling pathways could be a source of novel targets for therapeutic intervention of ocular diseases that have an angiogenic component. 

## 6. Conclusions

Despite years of study we still do not fully understand how *Bacillus anthracis* causes death. We do know that LeTx plays a critical role in the pathogenesis of the disease, and that the proteolytic function of LF is essential for LeTx activity. Further insight into the function of LeTx, and the role of LF metalloprotease activity upon anthrax infection, can be gained by analyzing the effect of LeTx on other pathological conditions. In both tumor and retinal models, the primary effects of LeTx lead to vascular dysfunction that causes hemorrhage and decreased perfusion (Table 2). While LeTx is capable of affecting multiple cell types, its common effect on vascular function indicates one or more cell types that modulate endothelial function, such as macrophages, pericytes, or endothelial cells, may be critical targets in the pathogenesis of this disease. However, the specific role of MAPKK inhibition in these pathologies by LeTx is still not understood. 

**Table 2:** Effect and consequences of LeTx metalloprotease activity.

**Effect**	**Anthrax [16,21,24,97]**	**Tumor [32,38,64,68]**	**Retina [84]**
Macrophage activity	Altered phagocytosis, Septic shock-like syndrome	n.d.	n.d.
Cytokine release	mixed effects *in vitro* and *in vivo*	Largely depressed *in vitro* and *in vivo*	Largely depressed *in vitro* and *in vivo * sequelae-elevated VEGF
Vascular function	Hemorrhage, vascular permeability, hypoxia, hypotension	Decreased perfusion, hemorrhage, decreased mean vessel density	Decreased perfusion, block in branching morphogenesis
**Consequence**	**Hypotensive shock, death**	**Decreased tumor volume**	**Retinopathy**

*n.d.: not determined.*
